# Electrochemical ammonia synthesis by reduction of nitrate on Au doped Cu nanowires†

**DOI:** 10.1039/d3ra00679d

**Published:** 2023-03-28

**Authors:** Yuankang Zha, Min Liu, Jinlu Wang, Jiyu Feng, Daopeng Li, Dongnan Zhao, Shengbo Zhang, Tongfei Shi

**Affiliations:** a Key Laboratory of Materials Physics, Institute of Solid State Physics, HFIPS, Chinese Academy of Sciences Hefei 230031 China tfshi@issp.ac.cn shbzhang@issp.ac.cn; b University of Science and Technology of China Hefei 230026 China

## Abstract

Electrochemical nitrate reduction reaction (NO_3_^−^RR) to synthesize valuable ammonia (NH_3_) is considered as a green and appealing alternative to enable an artificial nitrogen cycle. However, as there are other NO_3_^−^RR pathways present, selectively guiding the reaction pathway towards NH_3_ is currently challenged by the lack of efficient catalyst. Here, we demonstrate a novel electrocatalyst for NO_3_^−^RR consisting of Au doped Cu nanowires on a copper foam (CF) electrode (Au–Cu NWs/CF), which delivers a remarkable NH_3_ yield rate of 5336.0 ± 159.2 μg h^−1^ cm^−2^ and an exceptional faradaic efficiency (FE) of 84.1 ± 1.0% at −1.05 V (*vs.* RHE). The ^15^N isotopic labelling experiments confirm that the yielded NH_3_ is indeed from the Au–Cu NWs/CF catalyzed NO_3_^−^RR process. The XPS analysis and *in situ* infrared spectroscopy (IR) spectroscopy characterization results indicated that the electron transfer between the Cu and Au interface and oxygen vacancy synergistically decreased the reduction reaction barrier and inhibited the generation of hydrogen in the competitive reaction, resulting in a high conversion, selectivity and FE for NO_3_^−^RR. This work not only develops a powerful strategy for the rational design of robust and efficient catalysts by defect engineering, but also provides new insights for selective nitrate electroreduction to NH_3_.

## Abstract 2

Electrochemical nitrate reduction reaction (NO_3_^−^RR) to synthesize valuable ammonia (NH_3_) is considered as a green and appealing alternative to enable an artificial nitrogen cycle.

## Graphical abstract

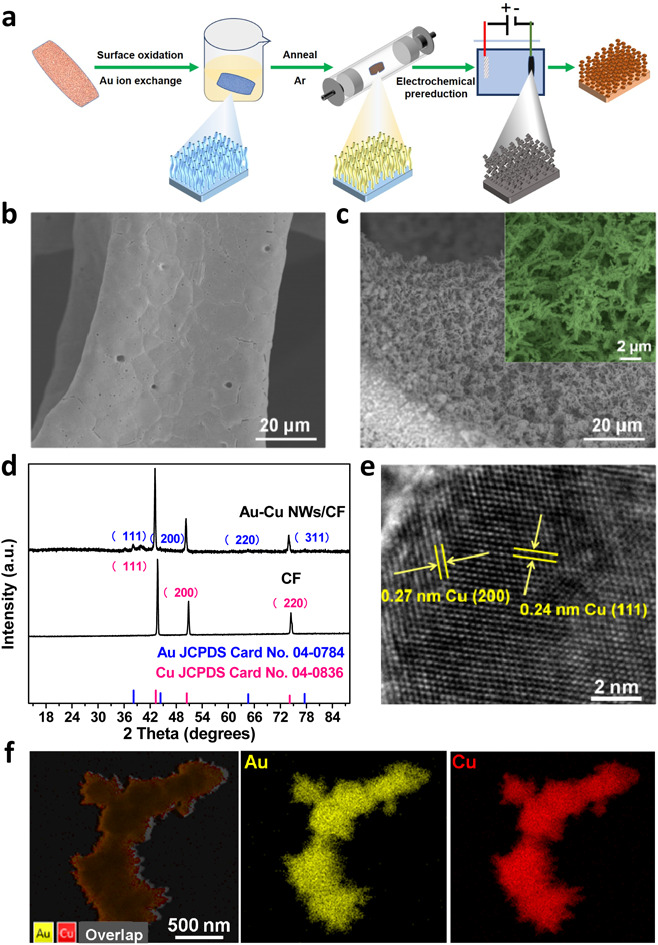

Ammonia (NH_3_) is not only an essential chemical and the cornerstone of the large and ever-growing fertilizer industry, but also considered as an important energy storage medium and carbon-free energy carrier.^1–4^ Currently, most of the ammonia synthesis in the world is implemented *via* the Haber–Bosch process, which consumes about 5.51 EJ of energy every year (∼38 GJ/*t*_NH_3__) and emits over 450 million metric tons of CO_2_ (∼2.9 *t*_CO_2__/*t*_NH_3__), this is because the process requires substantial driving force and hydrogen gas (*e.g.*, H_2_), which is produced from natural gas or coal through steam reforming, accounting for about half of CO_2_ emissions in the entire process.^5–8^ Nitrogen gas (N_2_) from air was identified as one major nitrogen source for this renewable route *via* electrochemical nitrogen reduction reaction (NRR), however, the faradaic efficiency (FE) is greatly hampered by the high dissociation energy of N≡N tripe bond (941 kJ mol^−1^) and poor solubility of N_2_ in electrolytes and the competitive reaction of H_2_ evolution.^9–11^ While exciting progresses in NRR catalyst development have been made, in many cases it is still challenging to firmly attribute the detected NH_3_ to NRR process rather than contaminations due to the extremely low NH_3_ production rate (mostly <200 μg h^−1^ mg_cat._^−1^).^12,13^ Thus, developing a new route for ammonia synthesis under benign conditions is urgently desired.

It is common knowledge that, nitrate pollution in surface water and groundwater is widespread in the world.^14^ High concentrations of nitrate in aquatic ecosystems pose a serious threat to ecological balances and human health. To minimize such adverse effects, many approaches including biological denitrification,^15^ reverse osmosis,^16^ ion exchange,^17^ electrodialysis,^18^ membrane filtration,^19^ electrocatalytic denitrification^20–22^ and so on have been adopted to dispose of nitrate contamination to produce clean water, among them, electrocatalytic denitrification driven by “green” electricity generated from renewable resources is the most likely practical alternative, which can overcome these limitations. Compared with the NRR, the nitrate reduction reaction (NO_3_^−^RR) to NH_3_ is not limited by the low solubility of N_2_ in water environment and its thermodynamically more favourable because of lower dissociation energy of N=O bond (204 kJ mol^−1^) than the N≡N tripe bond (941 kJ mol^−1^).^23,24^ Therefore, it is an frontier field that needs in-depth study.

Herein, we utilized a facile three-step method to fabricate the Au doped Cu nanowires on a copper foam (CF) (denoted as Au–Cu NWs/CF) electrode for the selective nitrate electroreduction to ammonia. The Au–Cu NWs/CF sample exhibited an exceptional performance with the NH_3_ yield rate of 5336.0 ± 159.2 μg h^−1^ cm^−2^ and the FE of 84.1 ± 1.0% at −1.05 V (*vs.* RHE) for the electrocatalytic NO_3_^−^RR under neutral conditions. ^15^N isotopic labelling experiments were performed to confirm the origin of ammonia, which was quantified by both ^1^H nuclear magnetic resonance (NMR) spectra and colorimetric methods. The XPS analysis and *in situ* infrared spectroscopy (IR) spectroscopy characterization results indicated that the oxygen vacancies in Au–Cu NWs/CF can weaken the N–O bonding,^25^ moreover, the electron transfer between Cu and Au interface could inhibit the competitive reaction of the hydrogen evolution reaction (HER),^11^ resulting in high NH_3_ yield rate and FE of NO_3_^−^RR.

Fig. 1a shows the schematic illustration of the growth of the Au doped Cu nanowires on a copper foam electrode. As illustrated in Fig. 1a, Au–Cu NWs/CF can be prepared by a three-step method. In the first step, the Cu(OH)_2_ NWs/CF was prepared *via* a facile wet-chemical oxidation method. Subsequently, NWs/CF was directly immersed into 10 mM HAuCl_4_·3H_2_O solution for 12 h, dried at 60 °C under vacuum for 4 h, the Au–Cu(OH)_2_ NWs/CF was annealed under Ar atmosphere to obtain Au–CuO NWs/CF. Finally, the Au–Cu NWs/CF was obtained by *in situ* electrochemical reduction of the resultant Au–CuO NWs/CF. The scanning electron microscopy (SEM) images of CF (Fig. 1b) and Au–Cu NWs/CF (Fig. 1c) demonstrate that the nanowires have been successfully generated on CF. After cation exchange reaction with Au precursor and subsequent thermal treatment and electrochemical reduction, the morphology of nanowires was largely maintained on the Au–Cu NWs/CF with the diameters of ∼100 nm (Fig. 1c). Fig. 1d shows the X-ray diffraction (XRD) patterns of CF and Au–Cu NWs/CF samples. As shown, similar diffraction peaks at 2*θ* = 43.3°, 50.4° and 74.1° can be observed for these two samples, corresponding to (111), (200) and (220) plane of metallic Cu (JCPDS no. 04-0836), respectively.^26–28^ While besides of typical diffraction peaks of metallic Cu, the Au–Cu NWs/CF sample also displays the weak characteristic peaks of Au nanoparticles at 2*θ* = 38.2°, 44.3°, 64.6° and 77.5°, suggesting the formation of fcc Au phase on Cu nanowires with low loading content.^29^ The actual loading of Au was calculated to be 5.6 wt% by inductively couple plasma atomic emission spectroscopy (ICP-AES). High-resolution TEM (HR-TEM, Fig. 1e) images show the lattice fringes of 0.24 and 0.27 nm, corresponding to the (111) and (200) planes of Cu, respectively, in good accord with the XRD results.^27,28^ In addition, the corresponding element mapping analysis of Au–Cu NWs/CF reveals that Au was homogeneously dispersed over the whole Cu foam (Fig. 1f).

**Fig. 1:**
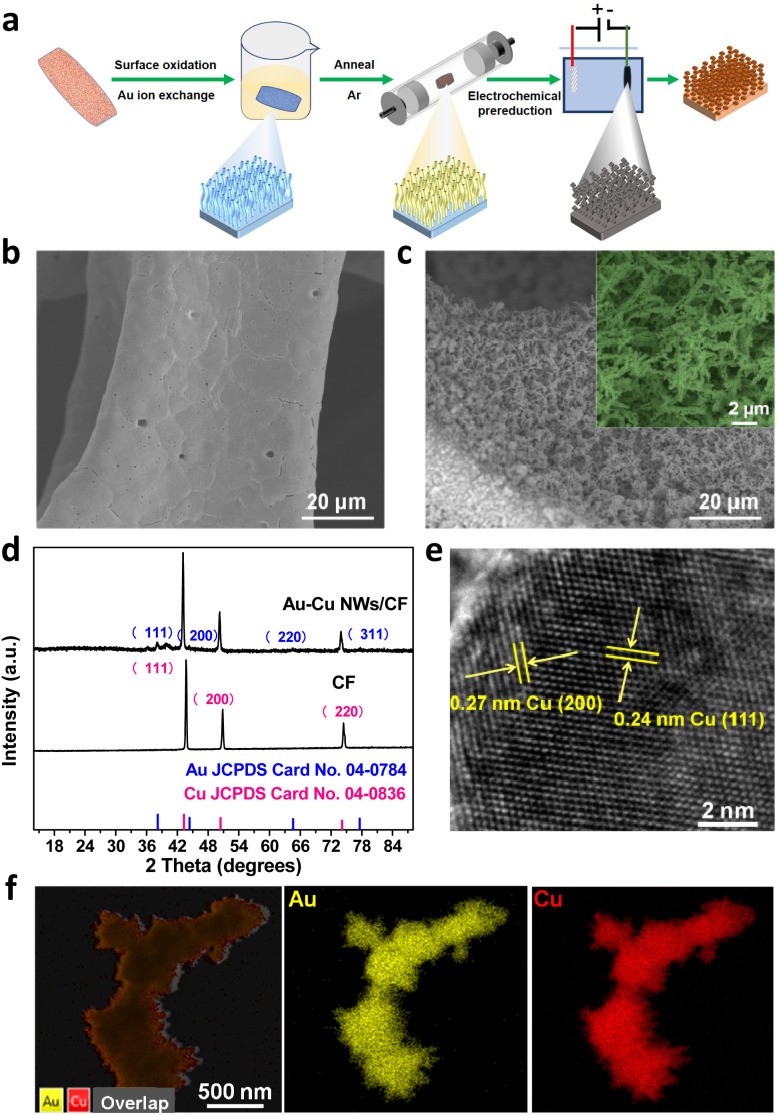
(a) Schematic illustration and corresponding structure of products. SEM images of (b) bare CF substrate and (c) Au–Cu NWs/CF. (d) XRD patterns for CF and Au–Cu NWs/CF. (e) HR-TEM image of Au–Cu NWs/CF. (f) EDS images of Au–Cu NWs/CF.

The X-ray photoelectron spectroscopy (XPS) measurement was performed to investigate the surface composition and valence state of Au–Cu NWs/CF. For comparison, we also performed the XPS characterization of CF sample. The XPS survey spectra and high-resolution XPS spectra of Au 4f verified the existence of doped Au in the Au–Cu NWs/CF (Fig. 2a and b). The high-resolution XPS spectra of Cu 2p in bare CF substrate is shown in Fig. 2c, where peaks of Cu 2p_3/2_ and Cu 2p_1/2_ appear at 932.5 and 952.3 eV.^26–28^ The two characteristic peaks confirms the presence of Cu^0^/Cu^1+^.^26–28^ Note that after Au doping, the binding energy of Cu 2p_3/2_ and Cu 2p_1/2_ shifted by 0.5 eV and 0.4 eV towards the lower binding energy of 932.0 and 951.9 eV in Au–Cu NWs/CF (Fig. 2c), due to the transfer of electrons between Cu and Au *via* chemical binding, which led to an increase in charge density and is conducive to electrocatalysis.^27,28^ Additionally, the new peak at binding energy of 934.2 eV was attributed to Cu^2+^ in Au–Cu NWs/CF. Based on previous reports,^27,28^ we further used Auger Cu LMM spectra to confirm the coexistence of Cu^0^ and Cu^1+^. It can be clearly observed in the Fig. S1† (ESI) that the Auger kinetic energy peak is wide and asymmetric in the range of 906 eV to 924 eV. The two asymmetric peaks with centers located at the position around 916.5 and 918.7 eV, 916.1 eV and 918.4 eV can be assigned to Cu^1+^ and Cu^0^ in the CF and Au–Cu NWs/CF, respectively.^27,28^ In the O 1s XPS spectra (Fig. 2d), 530.9 and 532.5 eV, 530.6 eV and 531.8 eV correspond to lattice oxygen and oxygen vacancy in the CF and Au–Cu NWs/CF, respectively.^26^ The significantly increased oxygen vacancy after doping is favourable for weakening the N–O bond and inhibiting the formation of by-products in the electrocatalytic nitrate reduction reaction, thereby improving the selectivity of ammonia.^25^

**Fig. 2:**
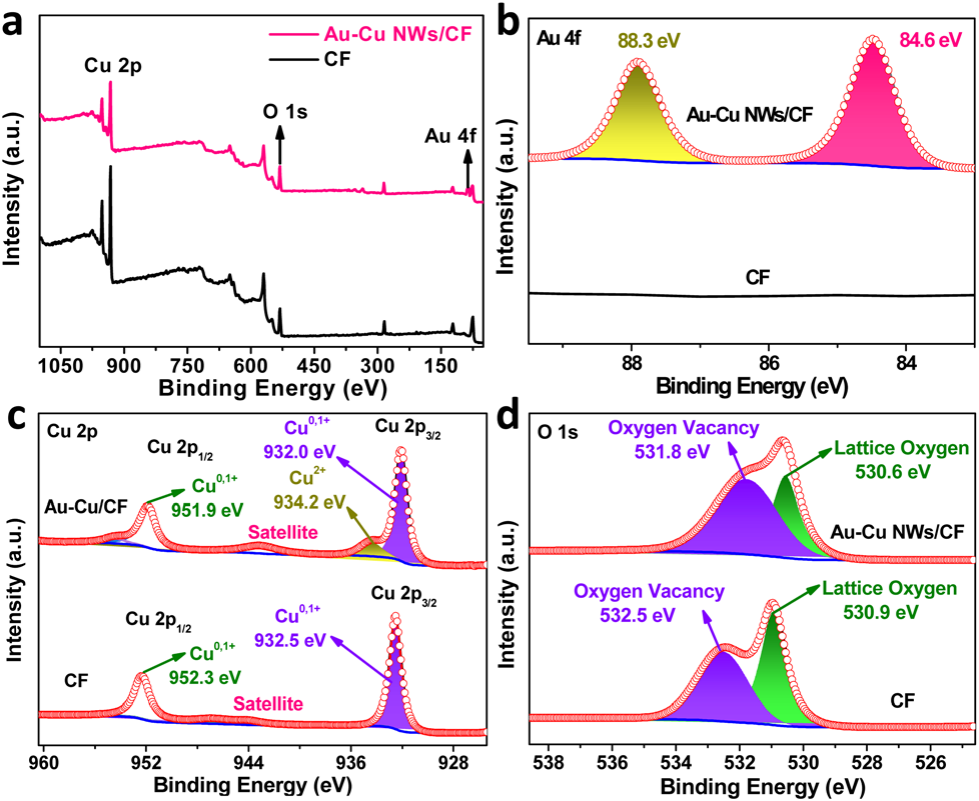
(a) Surface survey XPS spectra of Au–Cu NWs/CF and CF samples. High-resolution XPS spectra of (b) Au 4f, (c) Cu 2p and (d) O 1s in Au–Cu NWs/CF and CF samples.

We evaluated the NO_3_^−^RR performance of the as-fabricated Au–Cu NWs/CF catalysts in a 0.1 M Na_2_SO_4_ + 10.0 mM KNO_3_ solution (pH = 5.6) using a three-electrode configured two-compartment cell. In all experiments, the Au–Cu NWs/CF catalyst was used as working electrode, Ag/AgCl (saturated KCl solution) and Pt mesh were used as the reference electrode and counter electrode, respectively. Colorimetric methods were adopted determine the concentration of NO_3_^−^, NO_2_^−^ and NH_4_^+^ (Fig. S2, S3 and S4, ESI†). The liner sweep voltammetry (LSV) curves of Au–Cu NWs/CF electrocatalysts was conducted in 0.1 M Na_2_SO_4_ electrolytes with and without 10.0 mM KNO_3_. As shown in Fig. 3a, the current density increased obviously with the present of KNO_3_, suggesting that NO_3_^−^ in solution participated in the reduction reactions. Note that the LSV of Au–Cu NWs/CF tested in the presence of NO_3_^−^ exhibits a remarkable reduction peak at −0.6 V (*vs.* RHE), which may be due to the electrochemical reduction of NO_3_^−^. Chronoamperometry (CA) measurements of Au–Cu NWs/CF were conducted at different potentials for 2 h with continuous argon gas (Ar) bubbling. Fig. S5a† (ESI) shows the chronoamperometry curves at each given potential for 2 h electrolysis from −0.7 V to −1.1 V (*vs.* RHE). The concentration of NH_3_ product was measured using indophenol blue method (Fig. S5b, ESI†). The calculated NH_3_ yield rates and FEs based on three repeated experiments are given in Fig. 3b. It is worth noting that the Au–Cu NWs/CF achieved the highest NH_3_ yield rate (*R*_NH_3__) of 5336.0 ± 159.2 μg h^−1^ cm^−2^ and the FE of 84.1 ± 1.0% at −1.05 V (*vs.* RHE). The selectivity of NH_3_ (*S*_NH_3__) and *R*_NH_3__ show the same trend with the increase of potential, and highest *S*_NH_3__ was 90.6 ± 3.2% (Fig. 3c). In addition, the conversion of nitrate increases slowly with the increase of potential, and 100% conversion can be achieved at −0.95 V (*vs.* RHE) (Fig. 3d). When the potential further increased to −1.1 V (*vs.* RHE), the *R*_NH_3__ and *S*_NH_3__ decreased due to the competitive hydrogen evolution reaction (HER).^30^ Although the electrodynamic potential of NO_3_^−^ to NO_2_^−^ is higher than that of NO_3_^−^ to NH_3_, NO_2_^−^ is easily detected an main by-product of NO_3_^−^RR.^31^ As shown in Fig. S6† (ESI), few NO_2_^−^ is detected after electrocataytic reduction at −1.05 V (*vs.* RHE), further demonstrating the high selective reduction of nitrate to NH_3_. We also tested *R*_NH_3__ and FE of bare CF with 0.1 M Na_2_SO_4_ + 10.0 mM KNO_3_ solution at −1.05 V (*vs.* RHE) to exclude the influence of substrate. As shown in Fig. S7† (ESI), the highest *R*_NH_3__ and FE for bare CF were 1777.7 μg h^−1^ cm^−2^ and the FE of 49.9%, much lower than Au–Cu NWs/CF. The corresponding equivalent circuit diagrams of bare CF and Au–Cu NWs/CF are shown in Fig. S8† (ESI). The much lower ohmic resistances (*R*_s_) and charge-transfer resistance (*R*_ct_) from Au–Cu NWs/CF confirms its high electrical conductivity, which could be an important attribute for the achieved high *R*_NH_3__ and FE.

**Fig. 3:**
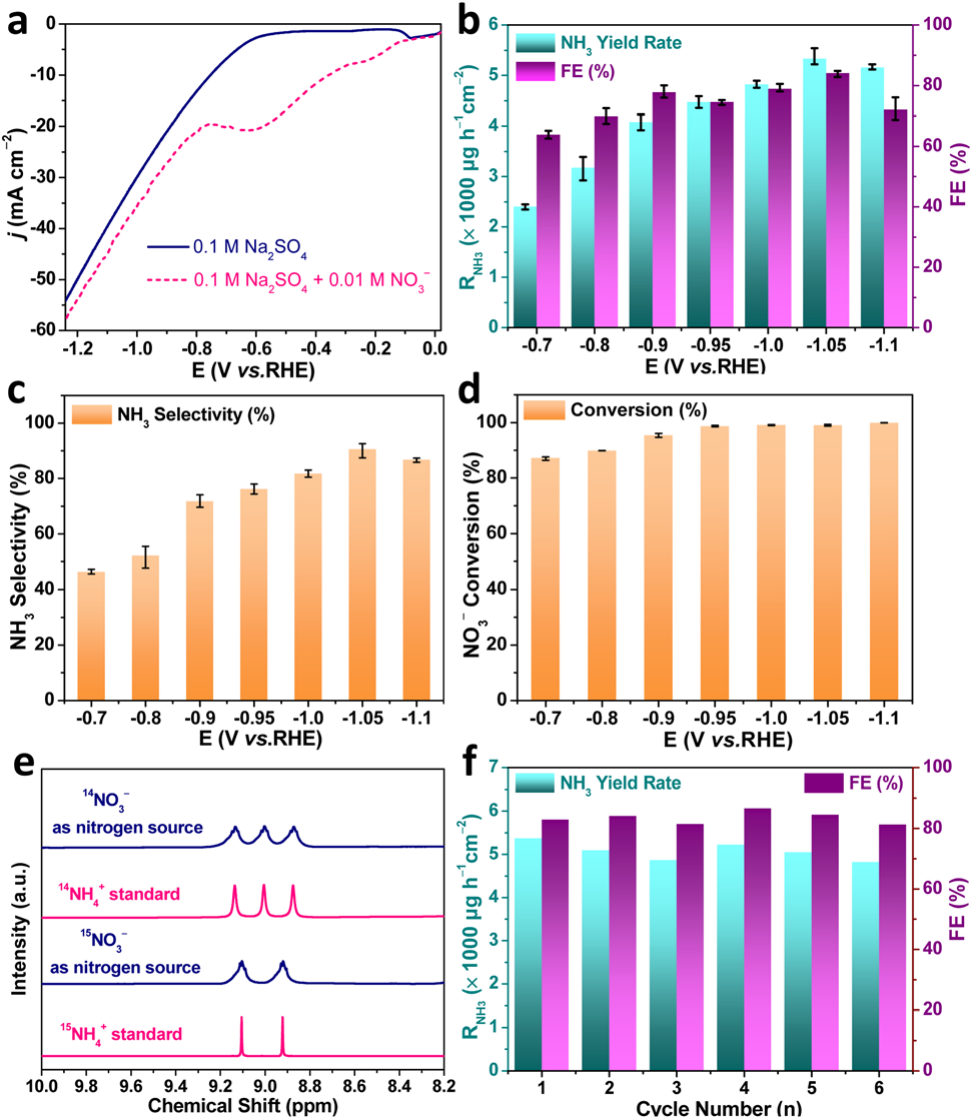
(a) LSV curves of Au–Cu NWs/CF catalyst in 0.1 M Na_2_SO_4_ and 0.1 M Na_2_SO_4_ + 10.0 mM KNO_3_ solution. (b) NH_3_ yield rate and faradaic efficiency of Au–Cu NWs/CF catalyst obtained at different potentials for 2 h NO_3_^−^RR measurement. (c) NH_3_ selectivity and (d) NO_3_^−^ conversion of Au–Cu NWs/CF catalyst obtained at different potentials. (e) ^1^H NMR spectra of Au–Cu NWs/CF catalyst using ^14^NO_3_^−^/^15^NO_3_^−^ as nitrogen source for NO_3_^−^RR and standards (^14^NH_4_)_2_SO_4_/(^15^NH_4_)_2_SO_4_. (f) Recycling tests for Au–Cu NWs/CF catalyst during NO_3_^−^RR at −1.05 V (*vs.* RHE).

^15^N isotope labeling with ^1^H nuclear magnetic resonance (NMR) is usually required in NO_3_^−^RR experiments to confirm that the detected NH_3_ indeed originates from the electrochemical nitrate reduction to rule out contaminations. We carried out chronoamperometry measurement at −1.05 (V *vs.* RHE) for 2 h in the electrolyte with K^15^NO_3_ and K^14^NO_3_ as N source, respectively. As shown in Fig. 3e, when the electrolysis was carried out in solution with K^14^NO_3_, the ^1^H NMR spectra of the obtained products displayed typical peaks of ^14^NH_4_^+^. In contrast, when the K^15^NO_3_ was used as nitrogen source, the ^1^H NMR spectra showed typical double peaks of ^15^NH_4_^+^. Such results indicated that the produced NH_3_ was entirely derived from the nitrate in the electrolyte, rather than from the contaminations. Meanwhile, the electrolyte without nitrate addition is also tested. The electrochemical measurement in blank 0.1 M Na_2_SO_4_ electrolyte produced ignorable NH_3_ (Fig. S9, ESI†), further confirming that the produced NH_3_ orginated from nitrate electroreduction. The durability of the Au–Cu NWs/CF electrocatalyst for NO_3_^−^RR was subsequently assessed by consecutive recycling electrolysis at −1.05 V (*vs.* RHE), no noticeable decay in the cathodic current density and UV-vis absorptions (Fig. S10, ESI†). As shown in Fig. 3f, the *R*_NH_3__ and FE are stable after 6 consecutive recycling tests, indicating the good durability of Au–Cu NWs/CF.

After electrolysis, the high-resolution Au 4f and Cu LMM XPS spectra were carried out to analyze the electronic properties of Au–Cu NWs/CF before and after NO_3_^−^RR measurement (Fig. S11, ESI†). Interestingly, after NO_3_^−^RR, the Au 4f_7/2_ shifted slightly to a lower binding energy by 0.2 eV after electrolysis (Fig. S11a, ESI†). Similarly, the Auger peak of Cu^2+^ shifted to a lower binding energy by 0.3 eV, while the Auger peaks of Cu^0^ and Cu^1+^ shifted to the higher binding energy by 0.2 eV and 0.3 eV after electrolysis (Fig. S11c, ESI†), indicating the existence of charge transfer between Au, Cu^2+^ and Cu^0,+1^ during NO_3_^−^RR process. Inaddition, the oxygen defect increased significantly after electrolysis (Fig. S11d, ESI†). In a word, the high electronic density of Cu^0^ and oxygen vacancy decreased the reduction reaction barrier and inhibited the generation of hydrogen in the competitive reaction, resulting in a high conversion, selectivity and FE of Au–Cu NWs/CF for NO_3_^−^RR.^31,32^

To gain a deeper understanding of the NO_3_^−^RR mechanism over Au–Cu NWs/CF catalysts, we utilized *in situ* infrared spectroscopy (IR) spectroscopy characterization to detect intermediates and monitor the reaction. Fig. 4a display the *in situ* IR spectra of Au–Cu NWs/CF under various potentials. As shown, without the applied potential, there is no any infrared peak in the *in situ* IR spectra. In the investigated potential range from −0.7 to −1.1 V (*vs.* RHE), the new infrared bands at ∼1541 cm^−1^ was assigned to the −NO_*x*_ intermediates.^33,34^ In addition, the bending mode of –NH_2_ is also found at ∼1457 cm^−1^.^34,35^ Clearly, as the applied potential increased, the peak intensity of −NO_*x*_ intermediates and –NH_2_ gradually increased (Fig. 4a). Fig. 4b shows the *in situ* IR measurements for the NO_3_^−^RR at −1.05 V (*vs.* RHE). The IR intensity of the peaks at around 1457 cm^−1^ and 1541 cm^−1^, corresponding to –NH_2_ and −NO_*x*_ intermediates is increased obviously from 4 to 36 min, implying that the NO_3_^−^RR takes place gradually with reaction time under the given electrocatalytic conditions. Evidenced by the *in situ* IR results, the NH_3_ synthesis by NO_3_^−^RR is successfully achievable (Fig. S12, ESI†), supportable for the electrocatalytic experimental results aforementioned.

**Fig. 4:**
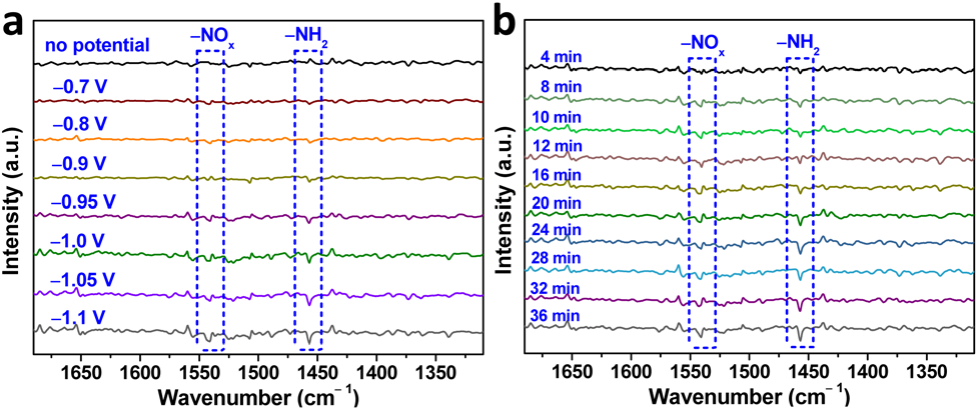
(a) *In situ* IR spectroscopy measurements under various potentials for Au–Cu NWs/CF in 1.0 M Na_2_SO_4_ + 10.0 mM KNO_3_ electrolyte. (b) *In situ* IR spectroscopy measurements of Au–Cu NWs/CF in 1.0 M Na_2_SO_4_ + 10.0 mM KNO_3_ electrolyte at −1.05 V (*vs.* RHE) for NO_3_^−^RR.

In conclusion, Au doped Cu nanowires on a copper foam electrode was synthesized *via* a facile three-step method, which further generated the oxygen vacancies in Au–Cu NWs/CF can weaken the N–O bonding, moreover, the electron transfer between Cu and Au interface could inhibit the competitive reaction, resulting in high conversion, selectivity and FE of Au–Cu NWs/CF for NO_3_^−^RR. The Au–Cu NWs/CF exhibited significantly enhanced NO_3_^−^RR activity with an NH_3_ yield rate of 5336.0 ± 159.2 μg h^−1^ cm^−2^ and the FE of 84.1 ± 1.0% at −1.05 V (*vs.* RHE) in neutral electrolyte. The *in situ* IR spectroscopy measurements confirm the successful realization of NH_3_ synthesis by NO_3_^−^RR over Au–Cu NWs/CF. Our work would be helpful to design and develop high-efficiency NO_3_^−^RR electrocatalysts for ambient electrosynthesis of ammonia.

## Conflicts of interest

There are no conflicts to declare.

## Supplementary Material

RA-013-D3RA00679D-s001
