# Supplementary material for: Electrochemical ammonia synthesis by reduction of nitrate on Au doped Cu nanowires
Source: RSC Adv. 2023 Mar 28;13(15):9839–44. doi: 10.1039/d3ra00679d (PMC10043758; doi:10.1039/d3ra00679d)
Supplement: RA-013-D3RA00679D-s001 [file RA-013-D3RA00679D-s001.pdf]

Electronic Supplementary Material (ESI) for RSC Advances.
This journal is © The Royal Society of Chemistry 2023

Electronic Supplementary Information

Electrochemical ammonia synthesis by reduction of nitrate on Au doped Cu nanowires

Yuankang Zha,[ab] Min Liu,[a] Jinlu Wang,[a] Jiyu Feng,[a] Daopeng Li,[a] Dongnan Zhao,[a] Shengbo Zhang,*[ab] and Tongfei Shi*[ab]

[a.] *Key Laboratory of Materials Physics, Institute of Solid State Physics, HFIPS, Chinese Academy of Sciences, Hefei 230031, China. E-mail: tfshi@issp.ac.cn, shbzhang@issp.ac.cn.*

[b.] *University of Science and Technology of China, Hefei 230026, China.*

Experimental Section

Materials. Hydrochloric acid (HCl)，ammonium persulfate ($((NH_4)_2S_2O_8)_2$, sodium hydroxide (NaOH)，ethanol (CH_3CH_2OH)，acetone (C_2H_6O)，potassium nitrate (KNO_3)，anhydrous sodium sulfate (Na_2SO_4) were purchased from Sinopharm Chemical Reagent Co., Ltd. (China), Gold chloride trihydrate ($HAuCl_4 \cdot 3H_2O$) was purchased from Aladdin Chemical Reagent CO., Ltd. (China)，Cu foam was purchased from Kunshan Shengshi Jingxin New Material Co., Ltd。

Catalyst synthesis. In a typical procedure, 1×2 cm^2 copper foam was ultrasonically cleaned in hydrochloric acid, acetone and ethanol for 10 minutes to clean the surface. Next, the washed Cu foam was soaked in 0.1 M $((NH_4)\ _2S_2O_8)_2$ with 2.5 M NaOH under ambient conditions for 20 min to oxidize Cu into $Cu(OH)_2$ nanowire. The synthesized $Cu(OH)_2$ nanowire was washed with deionized water and soaked in 10 mM $HAuCl_4 \cdot 3H_2O$ solution under ambient conditions for 12 h. Next, the product was dried at 60 °C in an oven for 4 h and then annealed in a flowing Ar atmosphere at 250 °C for 4 h to convert it into CuO doped with Au. After annealing, an in situ electrochemical pre-reduction step was performed, which was performed with the chronopotentiometry method at 100 mA cm^{-2} for 12 h, followed by 700 mA cm^{-2} for 1 h to obtain the final Cu foam doped with Au.

Characterization. Scanning electron microscope (SEM) images ware obtained by a field emission scanning electron microscope (FESEM SU8020).The powder X-ray diffraction (XRD) was obtained by X-ray diffracto meter (Philips X-Pert Super). The Kα ray source was a Cu target (λ=0.15418nm). X-ray photoelectron spectroscopy (XPS) was obtained by X-ray spectrometer (ESCALAB 250Xi).Transmission electron microscope (TEM) images and energy dispersive X-ray spectrum (EDS) images ware obtained by a field emission transmission electron Microscope (FETEM Tecnai G2 F20). The ^{1}H NMR (nuclear magnetic resonance) spectra were obtained using superconducting Fourier transform nuclear magnetic resonance spectrometer (Bruker Avance-400).

Electrochemical measurements. Electrocatalytic measurements were made in H-

type cells separated by proton exchange membranes, using the CHI 660E electrochemical Workstation (CH Instruments, Inc.) to record the electrochemical reactions. The synthesized Cu foam doped with Au was directly (effective area 1×1 cm^2)，Ag/AgCl (3 M) and Pt mesh were used as working electrode，reference electrode and counter electrode respectively. There was 70 ml electrolyte (0.1 M Na_2SO_4 + 10 mM KNO_3) evenly distributed in both cathode and anode chambers. Linear sweep voltammetry (LSV) was performed at a rate of 10 mV s^{-1}. The constant potential was tested for 2 h at different potentials. In this work, all measured potentials (*vs.* Ag/AgCl) were transformed into the potentials *vs.* reversible hydrogen electrode (RHE) based on the following equation:

$$E_{\mathrm{RHE}} = E_{\mathrm{Ag/AgCl}} + 0.059\mathrm{pH} + E^{\circ}{}_{\mathrm{Ag/AgCl}}$$

Determination of ammonia: Firstly, diluted electrolyte simple to the detection range (added 0.1 mL electrolyte to a 10 mL colorimetric tube containing 9.9 mL deionized water). Subsequently, 5 00 μL oxidizing solutions, 100 μL colour solution and 100 μL catalytic solution ware added into the colorimetric tube in turn. The oxidizing solution was formed by mixing 5 mL of sodium hypochlorite stock solution and 45 mL of 2 M sodium hydroxide solution. The colour solution contained 0.4 M sodium salicylate and 0.32 M sodium hydroxide. The catalytic solution was an aqueous solution with a concentration of 10g/L $Na_2[Fe(CN)_5NO]\cdot 2H_2O$. After the placement of 1 h at room temperature, the absorbance measurements were performed at wavelength of 697.5 nm. The obtained calibration curve was used to calculate the ammonia concentration.

Determination of NO_3^-: Firstly，diluted electrolyte simple to the detection range (added 1ml electrolyte to a 10ml colorimetric tube containing 9 mL deionized water). Subsequently, 200 μL 1 M hydrochloric acid and 20 μL of 0.8wt% sulfamic acid solution were added into the colorimetric tube in turn. The final absorbance is calculated using the following formula: $A = A_{220nm} - 2A_{275nm}$. The obtained calibration curve was used to calculate the nitrate concentration.

Determination of NO_2^-: Firstly, diluted electrolyte simple to the detection range

(added 0.1 mL electrolyte to a 10 mL colorimetric tube containing 9.9 mL deionized water). Subsequently，200 μL colour solution was added into the colorimetric tube. Colour solution was a mixture of p-aminobenzenesulfonamide (4.0 g), N-(1-naphthyl) ethylenediamine dihydrochloride (0.2 g), deionized water (50 mL) and phosphoric acid (10 mL, ρ = 1.685 g mL^{-1}). After shaking and standing for 20 minutes, the absorbance measurements were performed at wavelength of 540 nm. The obtained calibration curve was used to calculate the nitrite concentration.

Calculations.

The equation of NH_3 yield rate:

$$R(\mathrm{NH_3})(\mu\mathrm{g\,h^{-1}\ cm^{-2}}) = \frac{\mathrm{C(NH_4^+ - N)(\mu g\,mL^{-1})} \times V(\mathrm{mL}) \times 17}{t(\mathrm{h}) \times 14}$$

where R (NH_3) is the ammonia yield rate; C (NH_4^+–N) is the measured mass concentration of NH_4^+–N; V is the electrolyte solution volume; t is the reaction time; 14 is the molar mass of NH_4^+–N atom; 17 is the molar mass of NH_3 molecules.

The equation of Faradaic efficiency:

$$FE(\mathrm{NH_3})(\%) = \frac{8 \times n(\mathrm{NH_3})(\mathrm{mol}) \times F}{Q} \times 100\%$$

where F is the Faradaic constant (96485.34); Q is the total charge during the NO_3^-RR.

The equation of NO_2^- yield rate:

$$R(\mathrm{NO_2^-})(\mu\mathrm{g\,h^{-1}\ cm^{-2}}) = \frac{C(\mathrm{NO_2^-} - \mathrm{N})(\mu\mathrm{g\,mL^{-1}}) \times V(\mathrm{mL}) \times 46}{t(\mathrm{h}) \times 14}$$

where R (NO_2^-) is the ammonia yield rate; C (NO_2^-–N) is the measured mass concentration of NO_2^-–N; V is the electrolyte solution volume; t is the reaction time; 14 is the molar mass of NO_2^-–N atom; 46 is the molar mass of NO_2^- molecules.

The equation of Faradaic efficiency:

$$FE(\mathrm{NO_2^-})(\%) = \frac{2 \times n(\mathrm{NO_2^-})(\mathrm{mol}) \times F}{Q} \times 100\%$$

where F is the Faradaic constant (96485.34); Q is the total charge during the NO_3^-RR.

$^{15}N_2$ isotope labelling experiments. The ^{15}N isotopic labeling experiments was conducted using $K^{15}NO_3$ (99 atom % ^{15}N) as electrolyte in 0.1 M Na_2SO_4 with identical experimental procedure as that of $Na^{14}NO_3$ experiments. The ^1H NMR (nuclear magnetic resonance) spectra were obtained using superconducting Fourier

transform nuclear magnetic resonance spectrometer (Bruker Avance-400).

Supplementary Figures

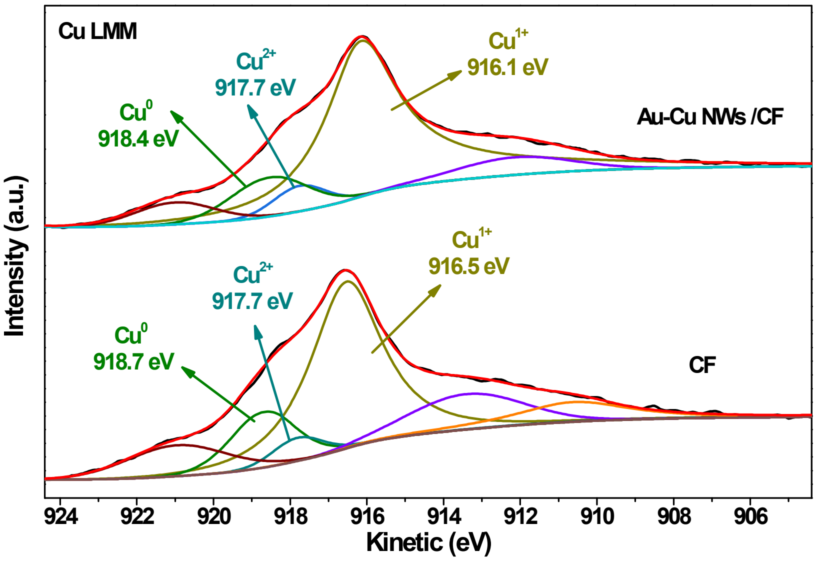

Fig. S1 Cu *LMM* XPS spectra of Au-Cu NWs/CF and bare CF substrate.

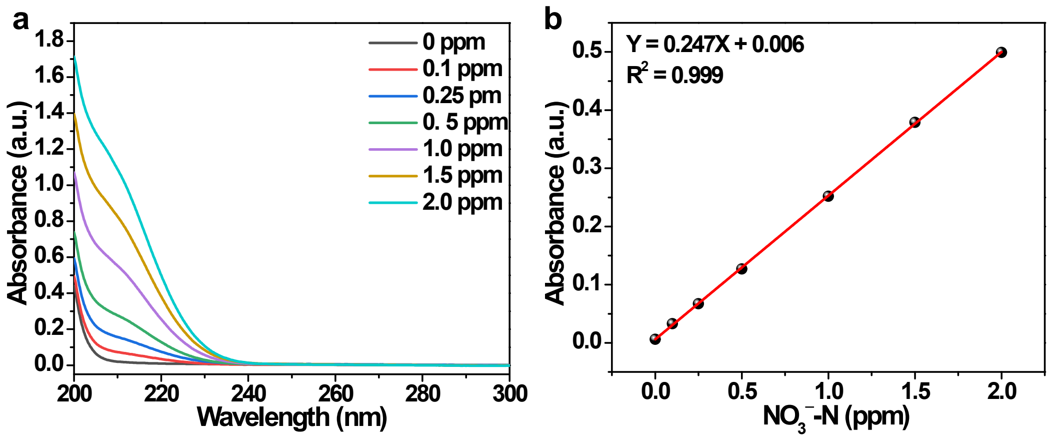

Fig. S2 (a) UV-Vis absorption spectra of various NO_3^--N concentrations (0, 0.1, 0.25, 0.5, 1.0, 1.5 and 2.0 ppm). (b) The calibration curve used for calculation of NO_3^--N concentration.

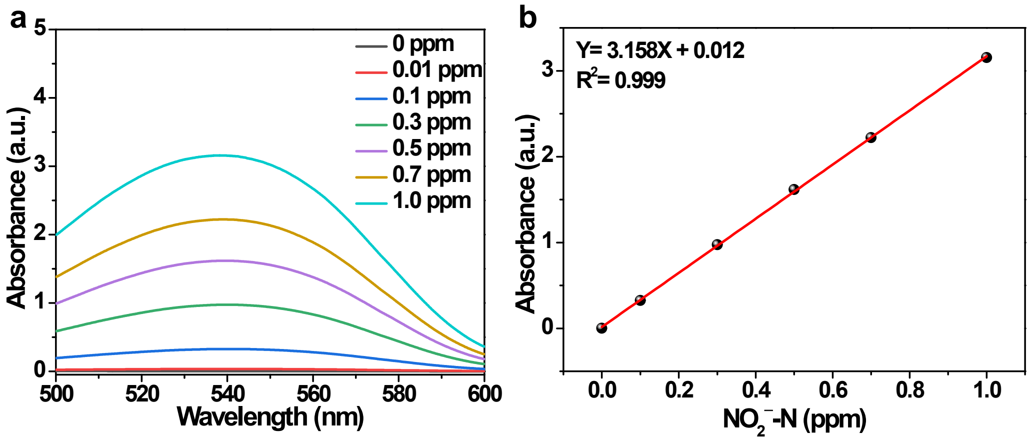

Fig. S3 (a) UV-Vis absorption spectra of various NO_2^--N concentrations (0, 0.1, 0.3, 0.5, 0.7 and 1.0 ppm). (b) The calibration curve used for calculation of NO_2^--N concentration.

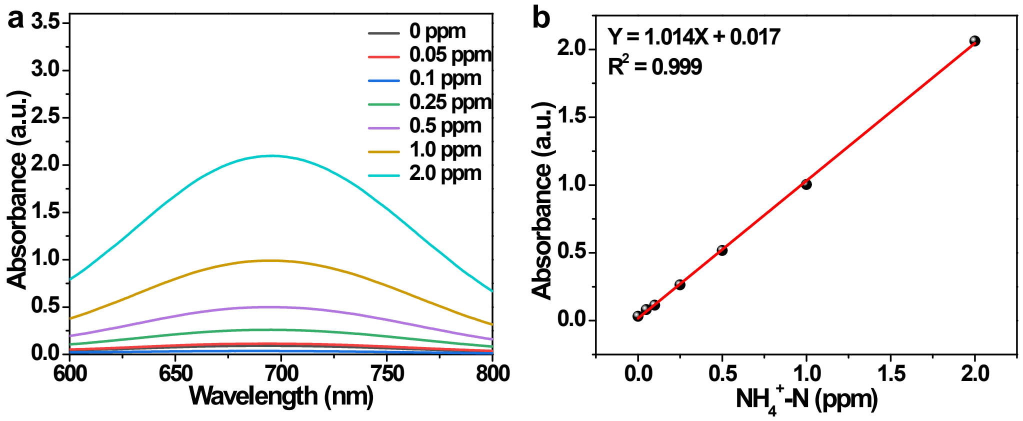

Fig. S4 (a) UV-Vis absorption spectra of various NH_4^+-N concentrations (0, 0.05, 0.1, 0.25, 0.5, 1.0 and 2.0 ppm). (b) The calibration curve used for calculation of NH_4^+-N concentrations.

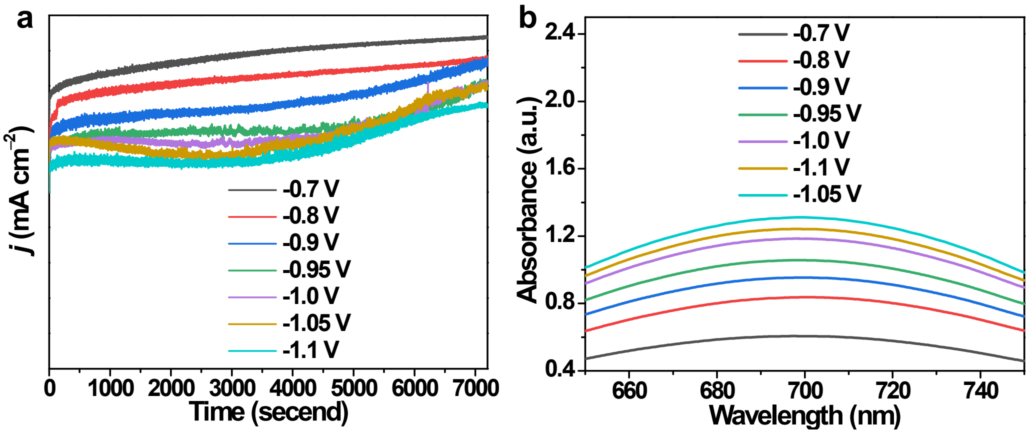

Fig. S5 (**a**) Time-dependent current density curves of Au-Cu NWs/CF catalysed NO_3^-RR at different potentials in 0.1 M Na_2SO_4 + 10.0 mM NO_3^- electrolyte over a 2 h period. (**b**) UV-Vis absorption spectra of the corresponding samples recorded in accordance with the indophenol blue method.

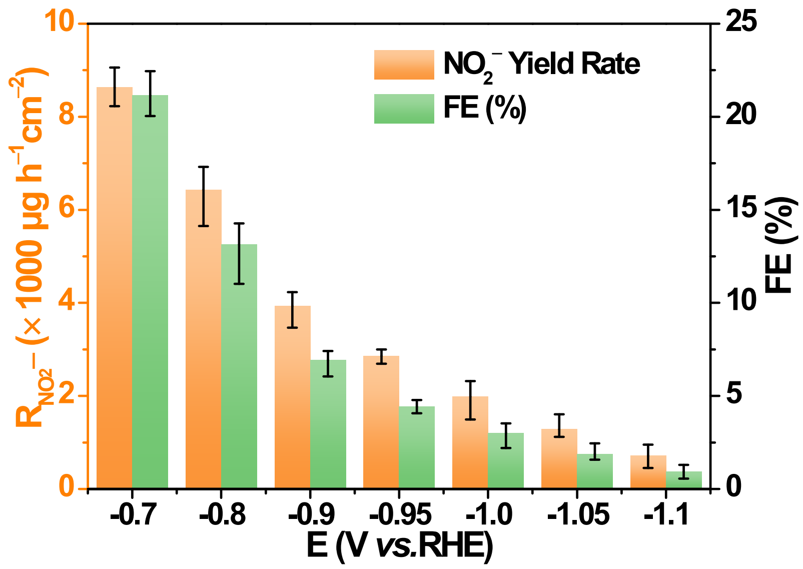

Fig. S6 NO_2^- yield rate and faradaic efficiency of Au-Cu NWs/CF catalyst obtained at different potentials for 2 h NO_3^-RR measurement.

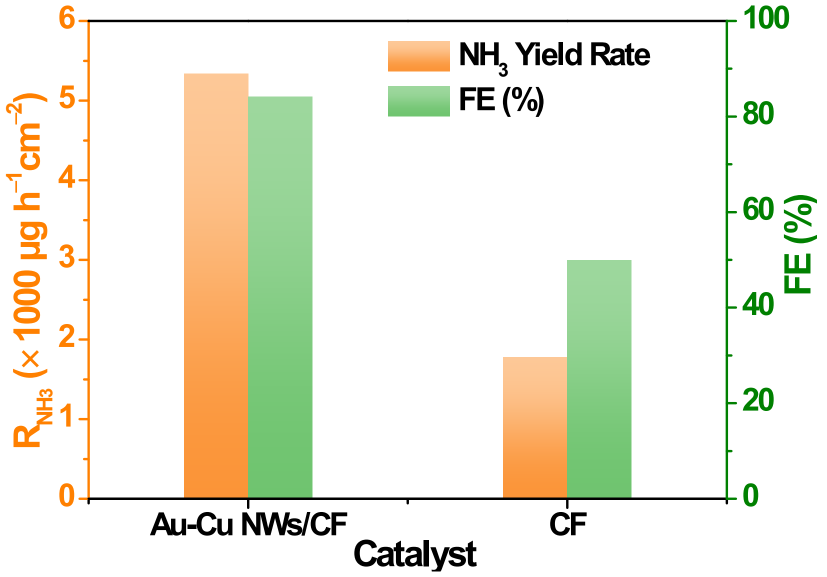

Fig. S7 NH_3 yield rate and faradaic efficiency of Au-Cu NWs/CF and bare CF catalysts obtained at −1.05 V (*vs*. RHE) for 2 h NO_3^-RR measurement.

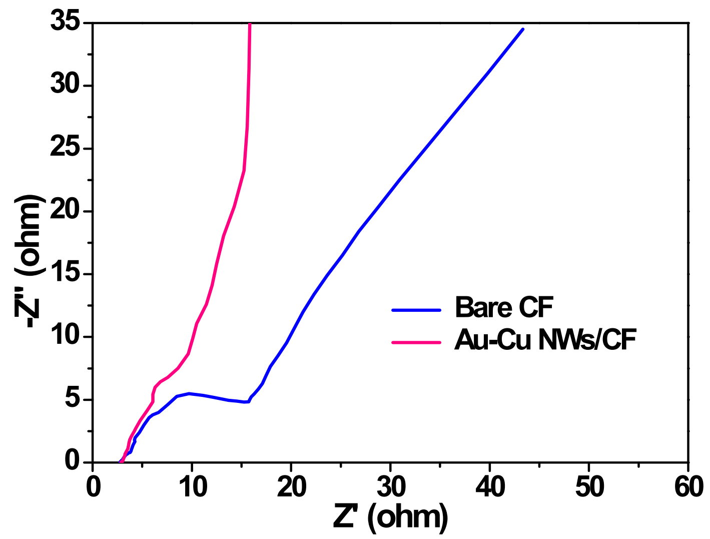

Fig. S8 EIS profiles of bare CF substrate and Au-Cu NWs/CF in 0.1 M Na_2SO_4 + 10.0 mM KNO_3 solution.

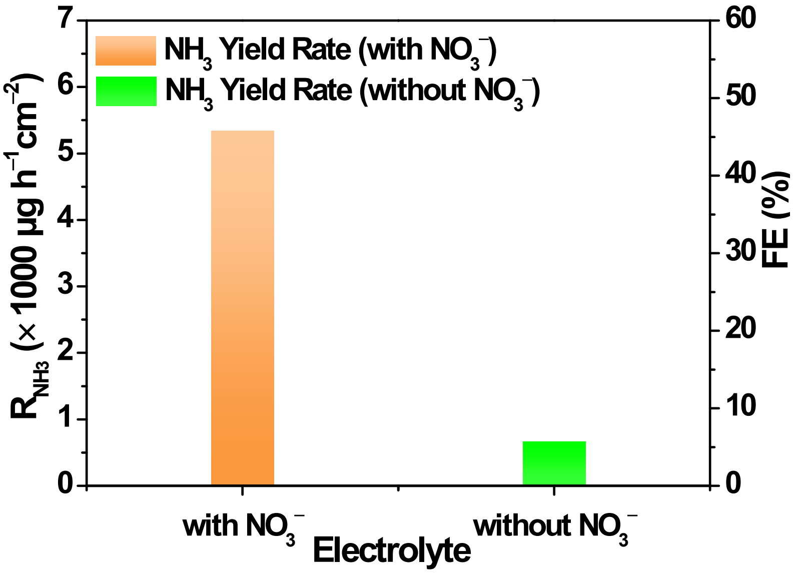

Fig. S9 NH_3 yield rate and faradaic efficiency over Au-Cu NWs/CF catalystsobtained in Na_2SO_4 electrolyte with and without nitrate at −1.05 V (*vs.* RHE) for 2 h NO_3^-RR measurement.

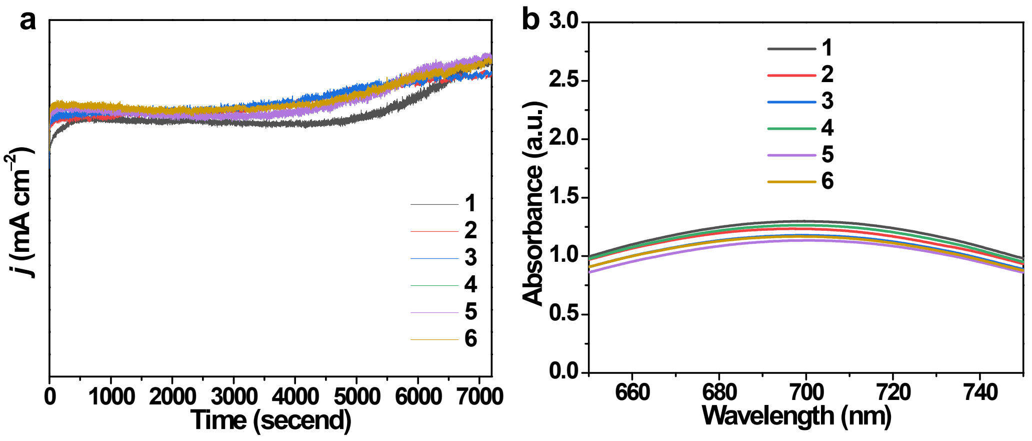

Fig. S10 (**a**) Cycling stability test of Au-Cu NWs/CF in 0.1 M Na_2SO_4 + 10.0 mM NO_3^- electrolyte at −1.05 V (*vs.* RHE) for 6 cycles with 2 h NO_3^-RR period per cycle. (**b**) UV-Vis absorption spectra of the corresponding samples recorded in accordance with the indophenol blue method.

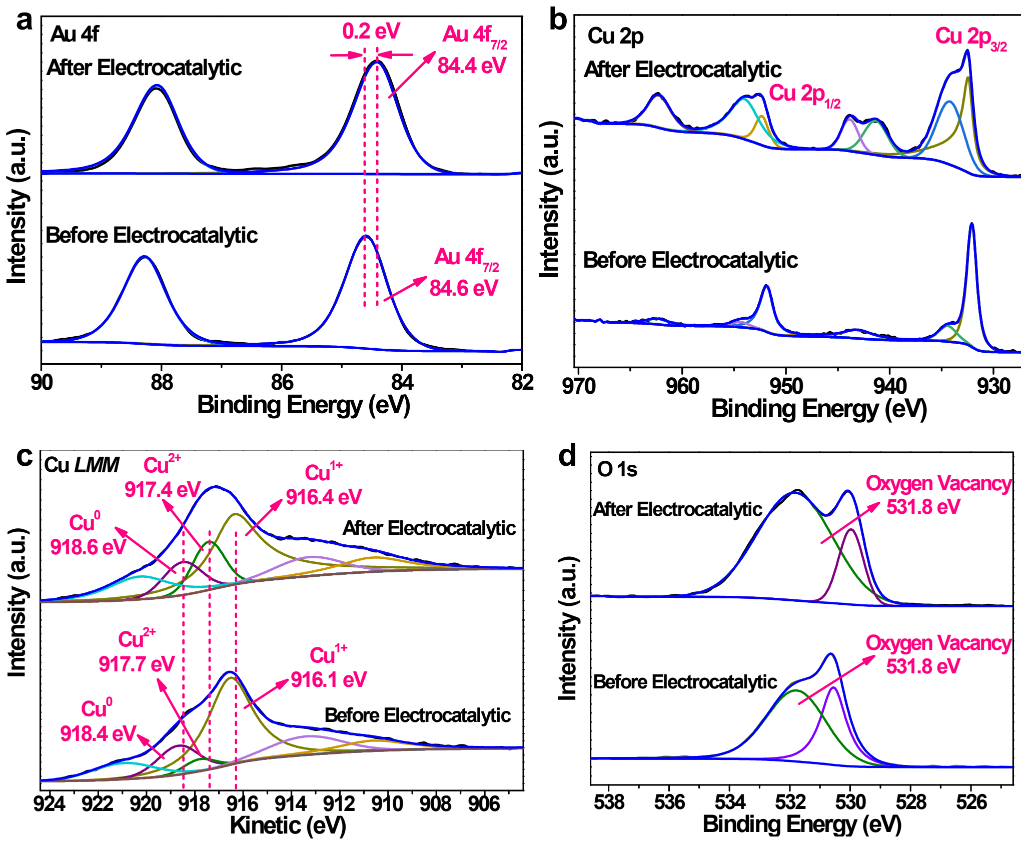

Fig. S11 High-resolution XPS spectra of (a) Au 4f, (b) Cu 2p, (c) Cu *LMM* and (d) O 1s in Au-Cu NWs/CF before and after NO_3^-RR.

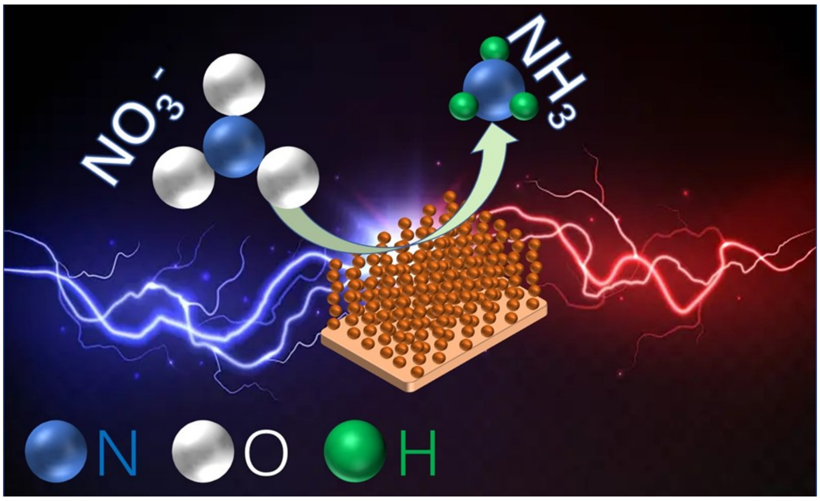

Fig. S12. Schematic diagram for NO_3^-RR.
